# The Stigmata of Maize and the Arenaria Rubra in Diseases of the Bladder

**Published:** 1879-11-20

**Authors:** 


### THE STIGMATA OF MAIZE AND THE ARENARIA RUBRA IN DISEASES OF THE BLADDER.

As the stigmata of maize are a very recent and as yet but little known addition to the materia medica, the following resume of the conclusions reached by Dr. Dufau, both from personal observation and from the reports of others, will undoubtedly prove interesting:

1. The stigmata of maize have a very marked, though not always a favorable action in all affections of the bladder, whether acute or chronic.

2. In acute traumatic cystitis, and also in gonorrhœal cystitis, they have a very marked diuretic action, but at the same time increase the pain; hence they should not be employed in these cases.

3. The best results have been obtained in cases of uric or phosphatic gravel, or chronic cystitis, whether simple or consecutive to gravel, and of mucous or muco-purulent catarrh. All the symptoms of the disease, the vesical pains, the dysuria, the excretion of sand, the ammoniacal odor, etc., etc., rapidly disappear under the influence of the medicine.

4. The retention of urine dependent on these various affections often disappears as improvement progresses, but the use of the sound must sometimes be continued, in order to empty the bladder completely.

5. The stigmata of maize have very often produced a cure after all the usual internal remedies had been tried in vain, or with only partial success. In other cases the ordinary methods of treatment, which had at first proved more or less entirely useless, became efficacious after the stigmata had been administered for a time, and had, as it were, broken the ground for them. Most frequently the stigmata alone sufficed for the cure, but still in some cases the effect was incomplete, and it was found that the treatment could be varied with benefit. Infections and irrigations of the bladder also proved useful adjuncts to the maize.

6. As the stigmata of maize are a very powerful, though at the same time entirely inoffensive diuretic, they have also been employed with the best results in cases of heart disease, albuminuria, and other affections requiring diuretics. Cases have been reported in which the urinary secretions were tripled and even quintupled in the first twenty-four hours, and others where the exhibition of the drug was continued for two or three months without the slightest untoward effect.

7. The best preparations of the stigmata are the extract and a syrup made from it. The decoction is unreliable and uncertain. The syrup, the usual dose of which is two or three teaspoonsful per diem, must be largely diluted, and for this purpose either hot or cold water, or a decoction of the stigmata may be used. The taste of this mixture is very agreeable. It should be given fasting.

Another remedy for diseases of the bladder that has lately begun to attract attention, is the arenaria rubra, a plant of the order of caryophylleœ. It is known in Algeria by the common name of sabline, and has long enjoyed repute in Malta and Sicily as a household remedy for the treatment of gravel and catarrh of the bladder. It seems to be useful in the same line of cases as the stigmata of maize, but as yet our knowledge concerning its powers and uses is very limited.—*Le Courier Medical*, May 3d and July 12th.

